# High Glucocorticoid Response to 24-h-Shift Stressors in Male but Not in Female Physicians

**DOI:** 10.3389/fendo.2017.00171

**Published:** 2017-07-18

**Authors:** Claudia Boettcher, Michaela F. Hartmann, Klaus-Peter Zimmer, Stefan A. Wudy

**Affiliations:** ^1^Division of Paediatric Endocrinology and Diabetology, Centre of Child and Adolescent Medicine, Justus Liebig University Giessen, Giessen, Germany; ^2^Steroid Research and Mass Spectrometry Unit, Centre of Child and Adolescent Medicine, Justus Liebig University Giessen, Giessen, Germany

**Keywords:** hypothalamic–pituitary–adrenal axis, gender, response to stressors, physicians, gas chromatography–mass spectrometry, urinary steroid metabolomics, glucocorticoid excretion

## Abstract

Physicians’ daily work is accompanied by emotional and physical stress, and 24-h shifts are considered to be a major stressor. Effects of stressors on the hypothalamic–pituitary–adrenal (HPA) axis can be evaluated by estimating the glucocorticoid excretion in urine samples. We characterized the impact of a 24-h working period on the urinary glucocorticoid excretion of physicians and focused on gender differences. 10 females and 12 male physicians collected 24-h urine samples during a 24-h shift (“on-duty”) and on a free weekend (“off-duty”) that were analyzed by gas chromatography–mass spectrometry. Urinary glucocorticoid excretion rates (GERs) were assessed by addition of the 24-h excretion rates per square meter body surface area for the seven major urinary cortisol and cortisone metabolites. Women showed generally lower glucorticoid excretion rates compared to men. Only male physicians had increased GERs on duty compared to off duty. As a measure of change between being on duty and off duty, the ratio GERs on duty/GERs off duty was significantly higher in males than in females. Thus, the 24-h shift stress factor generates diverging results between female and male subjects with activation of the HPA axis primarily in male physicians.

## Introduction

Physicians working in either hospitals or private practice are exposed to manifold stress factors, both physically and psychologically. Numerous examples are found in the literature stating a “stressed” condition of medical doctors worldwide (1–4). Possible effects of the doctoral stressor exposure are anxiety, sleep disturbances, depression, and burnout (5–8), culminating in a higher likelihood of suicide (9, 10) and—not to forget—of endangering patients (11, 12). Alongside many other factors, being “on call” and long working hours are especially recognized as burdening stressor elements (13–15).

The two main physiological systems that generally mediate the response to stressors are the—fast-reacting—sympathetic nervous system and the hypothalamic–pituitary–adrenal (HPA) axis, which reacts through release of glucocorticoids. Considering that particularly abnormalities in HPA function have been described *inter alia* in chronic inflammatory disorders, depression, and posttraumatic stress disorder (16–18), the integrity of the HPA axis appears to be most important for psychological, physiological, and physical health functioning.

Differences between men and woman regarding the reaction to stress seem possible and probable, e.g., studies in the setting of psychophysiological laboratories using the “Trier Social Stress Test” observed sex differences in response to this psychological stressor in salivary cortisol concentrations (19, 20) so called “stress-related” diseases occur mainly in one gender (21–24).

To assess the activation of the HPA axis, we have an elegant method available, namely urinary steroid metabolomics: with gas chromatography–mass spectrometry (GC–MS), excreted steroid metabolites in 24-h urine samples can be quantified in order to estimate the integrated output of steroid production (25, 26). As the sum of all urinary F (cortisol) metabolites reflects the 24-h-production rate of cortisol (27), this method is a useful tool to describe the glucocorticoid response to a prolonged stressor.

In the present study, we aimed to characterize the impact of a 24-h working period on the urinary glucocorticoid excretion of physicians and to investigate possible gender differences in response to this defined stressor.

## Materials and Methods

### Participants

Participants were recruited from the University Children’s Hospital Giessen. Eligible for our study were fulltime working physicians of all ages, healthy enough to participate in weekday 24-h shifts (regular working day from 8:00 a.m. to 5:00 p.m. in combination with night duty starting at 5:00 p.m., ending 8:00 a.m. the following morning). Collected demographic data included age, sex, height, weight, years of experience in pediatrics, and number of own children. The variables age and years of experience were categorized as ≤30 and >30 years of age and <5 and ≥5 years, respectively. In order to exclude physicians taking any medication with glucocorticoid effect, current medication was recorded. All participants gave written consent and the study was approved by the local ethics committee of the Justus Liebig University Giessen (no. 277/11).

### Urine Sample Collection

The participating physicians collected 24-h urine samples twice: once during a weekday 24-h shift (“on duty”) at the Children’s Hospital Giessen from 8:00 a.m. to 8:00 a.m. the following morning and again on a free and relaxing Sunday (“off duty”) at home, also from 8:00 a.m. to 8:00 a.m. The probands were instructed to catch their urine completely and each time they visited the toilet during the two 24-h periods. Additionally, they were requested to start the periods with an empty bladder, and to go to the toilet for a last catch at the very end of the 24 h. If a participant missed to collect one or more portions, he/she was asked to repeat the whole procedure during the next time he or she was “on” or “off duty.” In order to judge on the completeness of the collection, urinary creatinine was measured in the samples “on” as well as “off” duty. All samples were stored within a 1-h window after completion of collection at −20°C and thawed immediately before analysis.

During the urine sampling period “on duty,” hours of sleep (to the nearest 0.5 h), categorized as <4 and ≥4 h, and number of sleep disturbances, categorized as <3 and ≥3 interruptions were recorded.

### Urinary Steroid Hormone Analysis

Urinary steroid profiles were determined using GC–MS analysis according to the method described previously (26, 28, 29). In brief, free and conjugated urinary steroids were extracted by solid-phase extraction (Sep-Pak C18 cartridge; Waters Associates, Milford, MA, USA) out of an aliquot of 10 ml urine. The conjugated steroids were enzymatically hydrolyzed (type H-1 powdered Helix pomatia enzyme; Sigma Chemical Co., St. Louis, MO, USA) and recovered by Sep-Pak extraction. Known amounts of three internal standards (5α-androstane-3α,17α-diol, stigmasterol, and cholesteryl butyrate) were added to a portion of each extract before formation of methyloxime-trimethylsilyl ethers. Gas chromatography was performed using an Optima-1-fused silica column with helium as the carrier gas at a flow rate of 1 ml/min. Initial oven temperature was 80°C, held for 2 min; the temperature was then increased by 20°C/min to 190°C (1 min) and then increased by 2.5°C/min to 272°C. The gas chromatograph (Agilent 6890 Series GC; Agilent 7683 Series automatic liquid sampler; Agilent Technologies, Santa Clara, CA, USA) was directly interfaced to a mass selective detector (Agilent 5973N mass selective detector) operated in the selected ion monitoring mode.

To assess overall glucocorticoid excretion, the 24-h excretion rates for cortisol and its major metabolites tetrahydrocortisone, tetrahydrocortisol, 5α-tetrahydrocortisol, α-cortolone, β-cortolone, α-cortol, and β-cortol were added together and normalized to body surface area (BSA).

The ratio of glucocorticoid excretion rate (GER) off duty to GER on duty served as a robust artifice to compare changes between being on duty and off duty.

### Statistical Analysis

Descriptive analysis included calculation of mean and SD as well as median and range for continuous variables. Differences between males and females regarding age, years of experience, and BSA were tested by the unpaired *t*-test with Welch’s-correction. For comparison of the GERs, the paired and unpaired *t*-test (in case of normal distribution according to D’Agostino & Pearson omnibus normality test), or the Wilcoxon matched pairs signed rank test and the Wilcoxon–Mann–Whitney test were used. A *p*-value <0.05 was considered to indicate statistical significance. All statistical analyses were performed with GraphPad PRISM 6.02 (GraphPad Software, Inc., La Jolla, CA, USA).

## Results

### Description of Study Cohort

22 physicians of the Children’s Hospital Giessen agreed to participate in our study. Table 1 gives an overview of the population’s characteristics. BSA levels were in the normal range for all participants. Two male physicians had one, three other male colleagues two own children, whereas the remaining seven male and all the female probands were childless. All but one of the female doctors used oral or vaginal (NuvaRing^®^) contraception. Other medications used were levothyroxine (two females), bisoprolol (one male), cetirizine dihydrochloride (one male), and insulin (one male). The group of male doctors was slightly but significantly older and more experienced compared to the group of female physicians.

**Table 1:** Clinical characteristics of the population.

Variables	All probands (*n* = 22)	Females (*n* = 10)		Males (*n* = 12)
**Age (years)**				
Mean ± SD	32.4 ± 4.2	30.0 ± 2.7	*	34.3 ± 4.4
Median (range)	31.5 (27–41)	29.5 (27–35)		34.5 (27–41)
**Experience in pediatrics (years)**				
Mean ± SD	4.6 ± 3.1	3.1 ± 2.2	*	5.8 ± 3.2
Median (range)	4.0 (0.5–13.0)	2.5 (0.5–7.0)		5.0 (1.0–13.0)
**Body weight (kg)**				
Mean ± SD	70,94 ± 19.6	57.7 ± 10.4	**	81.4 ± 19.1
Median (range)	66.0 (44.0–120.0)	55.5 (44.0–77.0)		74.5 (62.0–120.0)
**Height (cm)**				
Mean ± SD	172.7 ± 8.8	165.6 ± 6.9	***	178.6 ± 5.0
Median (range)	174.0 (151.0–187.0)	167.5 (157.0–176.0)		177.5 (172.0–187.0)
**Body surface area (BSA) (m^2^)**				
Mean ± SD	1.8 ± 0.3	1.6 ± 0.2	***	2.0 ± 0.3
Median (range)	1.8 (1.4–2.5)	1.6 (1.4–1.9)		1.9 (1.8–2.5)
**Sleep interruptions on duty**				
Mean ± SD	2 ± 2	2 ± 2		2 ± 2
Median (range)	2 (0–7)	2 (0–6)		2 (0–7)
**Hours of sleep on duty**				
Mean ± SD	4.0 ± 1.3	3.7 ± 1.1		4.2 ± 1.4
Median (range)	4.0 (1.5–6.5)	3.8 (1.5–5.3)		4.3 (1.5–6.5)
**24-h urine volume on duty (ml)**				
Mean ± SD	2,026 ± 1,056	1,735 ± 1,006		2,268 ± 1,078
Median (range)	2,110 (650–4,860)	1,480 (650–4,000)		2,183 (810–4,860)
**24-h urine volume off duty (ml)**				
Mean ± SD	2,200 ± 772	2,339 ± 778		2,084 ± 780
Median (range)	2,165 (850–3,725)	2,275 (1,300–3,725)		1,925 (850–3,230)

*Significant differences (unpaired *t*-test with Welch’s-correction) between males and females concerning the variables age, experience in pediatrics and BSA*.

*p < 0.05.

**p < 0.01.

***p < 0.001.

### Urinary GERs

According to the non-significantly differing urinary creatinine values between “on” (mean 102.9 mg/dl ± 54.2 SD) and “off” duty (mean 80.0 mg/dl ± 42.6 SD), which were all within normal range, completeness of urine collection was demonstrated.

The GERs off duty in our cohort corresponded excellently with published references (30, 31).

**Figure 1:**
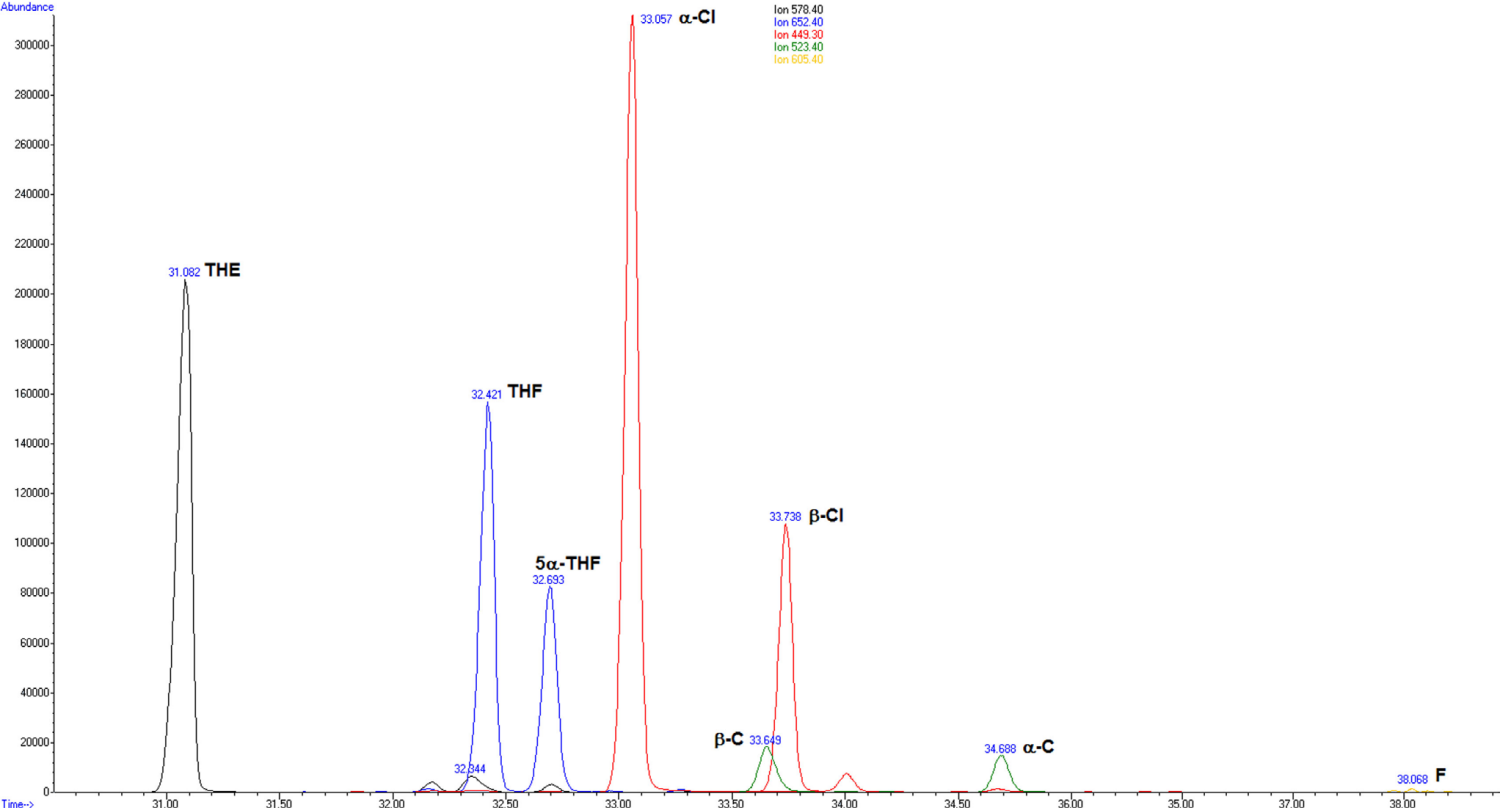
Merged extracted ion chromatograms for the glucocorticoids measured in the urine of a male proband on duty: *m*/*z* (mass to charge ratio) 578.4 tetrahydrocortisone (THE); *m*/*z* 652.4 tetrahydrocortisol (THF) and 5α-tetrahydrocortisol (5α-THF); *m*/*z* 449.3 α-cortolone (α-Cl) and β-cortolone (β-Cl); *m*/*z* 523.4 α-cortol (α-C) and β-cortol (β-C); and *m*/*z* 605.4 cortisol (F).

Exemplarily, Figure 1 shows merged extracted ion chromatograms of a physician on duty.

**Figure 2:**
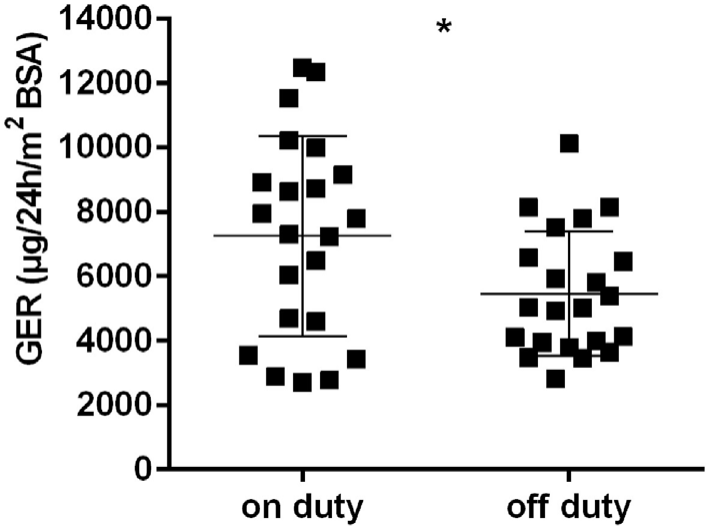
Glucocorticoid excretion rates [μg/24 h/m^2^ body surface area (BSA)] in all physicans, on and off duty; horizontal bars indicate mean and SD; *p < 0.01.

Considered as a whole, the group of physicians showed a marked GER rise in urine collected during a 24-h shift in comparison to a free weekend (*p* < 0.01) (Figure 2). Neither sorting the physicians by age (≤30 versus >30 years of age) nor by years of experience in pediatrics (<5 versus ≥5 years) resulted in significant differences for the GER on duty. The same was true for the stratification by number of sleep disturbances (<3 versus ≥3 interruptions) and by hours of sleep (<4 versus ≥4 h).

**Figure 3:**
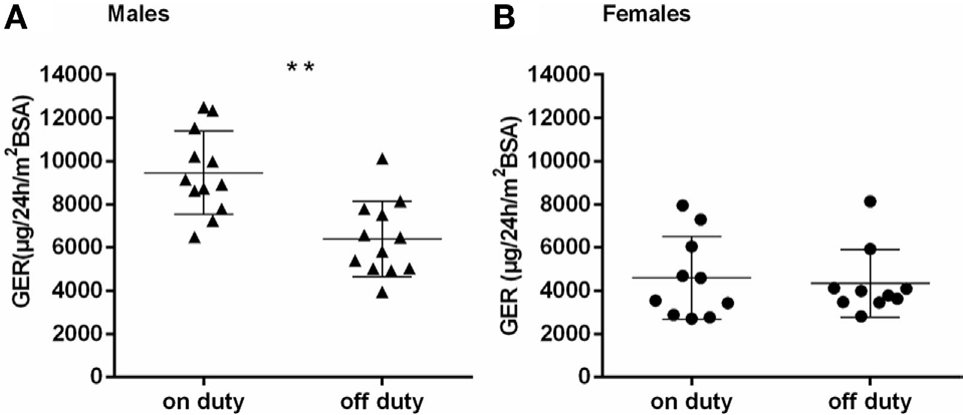
**(A,B)** Glucocorticoid excretion rates [μg/24 h/m^2 ^body surface area (BSA)] in males **(A)** and females **(B)**, on and off duty; horizontal bars indicate mean and SD; **p < 0.001.

**Figure 4:**
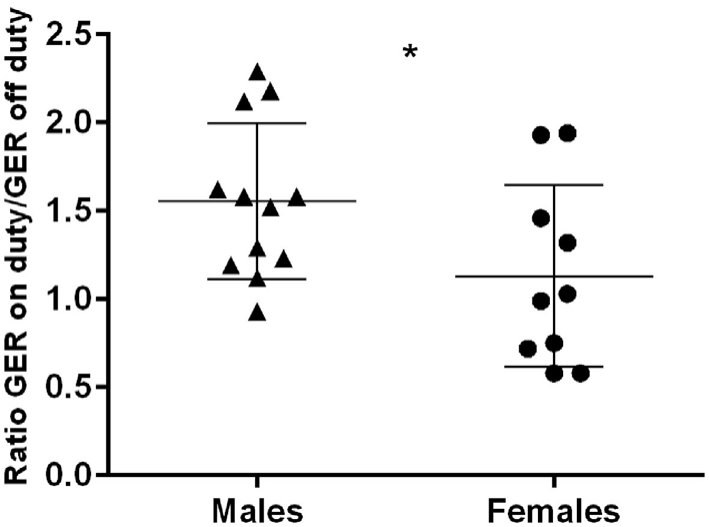
Ratio GER on duty/off duty in males and females; horizontal bars indicate mean and SD; *p < 0.05.

The GER separated by gender, on and off duty, are presented in Figures 3A,B. Female physicians had generally lower GER than male physicians, regardless of being on or off duty (*p* < 0.001 and *p* < 0.01, respectively). The group of male physicians presented with higher GER on duty than off duty (*p* < 0.001). No such effect could be detected in the female group. The number of sleep disturbances or hours of sleep differed not significantly between males and females. Regarding the ratio GER on duty/GER off duty, a significantly higher ratio could be seen in males than in females (*p* < 0.05) (Figure 4).

## Discussion

Many of the current studies concentrating on “stress research” with assessment of the HPA axis have employed salivary cortisol as a biomarker, taking advantage of its non-invasiveness and simplicity in sampling (32). In the salivary gland, cortisol is metabolized to cortisone by the action of 11β-hydroxysteroid dehydrogenase type 2, and cortisone is partly responsible for the variable interference observed in salivary cortisol immunoassays (33). Less frequent study-tools are 24-h urinary (free) cortisol or serum parameters whereas studies using urinary steroid metabolomics are rare. Therefore, despite limiting the comparability of our results by using GC–MS urinary steroid profiling, there are several advantages in favor of our method: the urine collection is non-invasive and independent of fixed time points for sampling (except for onset and end of the 24-h collection period), and hence practicable during 24-h shifts in a hospital. With this approach, we were able to obtain sample material covering the entire period of stressor exposure on duty; not only cortisol but *all* urinary glucocorticoid metabolites are analyzed and quantified by GC–MS, allowing the calculation of GER.

As psychological and physical stressors are capable of activating the HPA axis, two types of stressors are commonly used in the assessment of stressor-responsiveness: psychological tasks (e.g., delivering a public speech) or physiological tests (e.g., cold pressures test) (34, 35). In our study, the “stressor” (24-h shift) was a natural combination under “field conditions” with variable shares of both stressor types, therefore reflecting real-life conditions instead of laboratory settings.

It is well known—proven by urinary cortisol excretion measurements (26, 36–38) as well as stable-isotope tracer infusions (39)—that a general sexual dimorphism exists with higher cortisol excretion rates in men than in women. Our results with constantly lower GER in female physicians compared to male physicians confirm this knowledge. In contrast, four studies of one working group measuring salivary cortisol responses to psychosocial stress could not detect considerable pre-stressor gender differences in healthy subjects [summarized by Kirschbaum et al. (19)]. Methodological issues (salivary versus urinary samples, “snapshot” versus comprehensiveness of 24-h sampling) might explain those differences.

Considered in their entirety, our physicians showed marked adrenocortical activation in response to the 24-h stressor. However, this effect seems mainly owed the male participants, as—when divided into groups sorted by gender—only the male but not the female group of physicians showed significantly higher GER on duty compared to off duty. In a small group of solely male physicians, Vierhapper and Nowotny (40) saw comparable “male” results: eight internal medicine residents excreted considerably more urinary glucocorticoid metabolites during their work time (24-h shift) than on weekends. But concerning gender differences in response to various stressors, the results are inconsistent: in reaction to defined acute psychosocial stressors, women seem to show less ACTH and cortisol responses than men (41); responses to physical stressors appear not to differ between men and woman (42–44); and in a “real life” study of Marchand et al. (45), salivary cortisol levels increased linearly from non-working day to work day in a random sample of day-shift employees, with no significant gender differences.

What could explain that our female group did not react with a higher GER on duty than off duty like the male group did? The first explanation for this phenomenon would be that the women with children and running a family are equally stressed on a weekend than during a weekday 24-h shift. As none of the female physicians had children at the time of participating in our study whereas five of the male physicians lived with children, this discussion point can be ruled out. In a second step, we would consider hormonal influences as part of the menstrual cycle: different phases (follicular phase versus luteal phase) are known to be modulators of the cortisol stress response (41); but all but one of our female subjects of research used hormonal contraception, “equalizing” the women and making an effect of menstrual cycle phases unlikely. Hormonal contraception itself attenuates responses to stressors in terms of lowering (salivary) cortisol levels (46, 47). But this known effect cannot explain the women’s stressor response pattern in our study since it is an intraindividual comparison. Another argument could be that for the women “work replaced home as a haven”: in a recent study Damaske et al. (48) investigated the proposal that work may be less stressful than home. They revealed that salivary cortisol levels of employees—moderated by income and children at home—can indeed be even lower at work (not shift work) than at home. However, these carefully collected data applied not only to women but also to men.

One could assume that variable hours of sleep deprivation might contribute to an increase in the GER on duty. In fact, several recent studies applying up to 40 h of sleep deprivation showed increased levels of serum cortisol after the intervention (49, 50); however, Tobaldini et al. (51) failed to detect a significant difference in serum cortisol levels of 15 healthy residents (mixed group, 10 males and 5 females) before and after one night on call. In our study, when assigned to two respective groups (<3/≥3 sleep interruptions; <4/≥4 h of sleep), there was no significant difference in the GER on duty detectable.

The age of our male participants was higher than the female physicians’, and the males were more experienced in pediatrics. Therefore, we cannot deny the possibility that this might have contributed to the gender differences seen in the stress–response of our study: a study involving subjects up to 88 years of age suggested an effect of older age on salivary cortisol (52); a detailed review by Kudielka et al. (53) provided a controversial discussion of a possible age effect on cortisol stressor-responses, also pointing out a potential effect in elderly men—again based upon measurements in saliva. The age-range (27–41 years of age) of our physicians, however, was markedly narrower than in the study of Seemann et al. (52) mentioned above, and excluded the elder age. Regarding work experience, Dyrbye et al. (7) saw—with the instrument of questionnaires—in a large sample of physicians (men and women) from all specialty disciplines that compared to other career stages, the “middle career period” (11–20 years of practice) is a particularly challenging time in terms of burnout and satisfaction. With a median of 4.0 (range 0.5–13.0) years of experience, most of our study physicians stay clearly under this period.

In summary, our study has shown that the stressor 24-h shift is able to activate the HPA axis mainly in male, but not in female physicians, detectable by urinary steroid profiling. The further interpretation of our data and consecutively the implications remain speculative at the present moment: the findings could point to a certain female “stress resistance” regarding long shift work, or otherwise to a blunted physiological response toward this stressor. The “male” reaction with high glucocorticoid excretion on duty could cause long-term health problems like hypertension and coronary artery calcification (54, 55) or even result in a shorter lifespan in males (56).

## Ethics Statement

This study was carried out in accordance with the recommendations of the ethics committee of the Justus Liebig University Giessen with written informed consent from all subjects. All subjects gave written informed consent in accordance with the Declaration of Helsinki. The protocol was approved by the ethics committee of the Justus Liebig University Giessen (no. 277/11).

## Author Contributions

CB and SW substantially contributed to the conception and design of the work, CB drafted the work. CB, MH, and K-PZ are responsible for acquisition, analysis, and interpretation of the data. MH, SW, and K-PZ revised the work critically for important intellectual content. CB, MH, SW, and K-PZ gave final approval of the version to be published and agree to be accountable for all aspects of the work in ensuring that questions related to the accuracy or integrity or any part of the work are appropriately investigated and resolved.

## Conflict of Interest Statement

The authors declare that this article was written in the absence of commercial or financial relationships that could be interpreted as possible conflicts of interest.
